# Minimally invasive pelvic exenteration for gynaecological malignancies: the challenge of patients’ selection

**DOI:** 10.52054/FVVO.15.3.084

**Published:** 2023-09-24

**Authors:** N Bizzarri, V Chiantera, M Loverro, A Ercoli, G Vizzielli, G Scambia

**Affiliations:** UOC Ginecologia Oncologica, Dipartimento di scienze della salute della donna, del bambino e di sanità pubblica, Fondazione Policlinico Universitario A. Gemelli, IRCCS, Rome, Italy; Unit of Gynecologic Oncology, ARNAS “Civico – Di Cristina – Benfratelli”, Department of Health Promotion, Mother and Child Care, Internal Medicine and Medical Specialties (PROMISE), University of Palermo, Palermo, Italy; Department of Human Pathology of Adult and Childhood “G. Barresi”, Unit of Gynecology and Obstetrics, University of Messina, Messina, Italy; Department of Obstetrics, Gynecology, and Pediatrics, Obstetrics and Gynecology Unit, Udine University Hospital, DAME, Udine, Italy

## Abstract

Pelvic exenteration is a radical procedure representing a salvage option in patients with recurrent or persistent gynaecological malignancies. It can be performed with an open or minimally invasive approach. Different studies have demonstrated optimal peri-operative outcomes of minimally invasive pelvic exenteration with no survival difference when compared with an open approach. In this article, we discuss the importance and the challenge of patient selection for pelvic exenteration and more specifically for minimally invasive pelvic exenteration.

Pelvic exenteration is the salvage curative option for patients with recurrent or persistent gynaecological cancer after radiation therapy. It is a major radical procedure involving removal of two or more pelvic organs. (Sardain et al., 2015).

Different studies have assessed the feasibility (Pomel et al., 2003) and the peri-operative outcomes (Bizzarri et al., 2019) of pelvic exenteration performed with a minimally invasive approach. Some studies have reported better peri-operative morbidity in patients undergoing minimally invasive, compared with open pelvic exenterations (Bizzarri et al., 2019; Matsuo et al., 2021; Martínez et al., 2011). Very few studies focused on the survival outcomes of patients undergoing a minimal access approach (Martínez et al., 2011; Puntambekar et al., 2016).

As pelvic exenteration is associated with major post-operative complications in 18-27% (Tortorella et al., 2019; Bizzarri et al., 2023) and with post- operative death in 2% (Tortorella et al., 2019), the selection of patients becomes of paramount importance. Crucially, in studies where cases were selected based on favourable prognostic factors (central location, small size, no lymph-vascular space invasion (LVSI), no pelvic lymph node involvement, long time to recurrence after radiation therapy), the procedure was shown to be curative if negative surgical margins were achieved, with overall survival ranging between 48-64% (Schmidt et al., 2012; Chiantera et al., 2014).

More recently, surgical boundaries for a potentially curative radical surgery have been pushed. Particularly, laterally extended disease which reaches the pelvic side wall (Hockel, 2015) and even disease which involves one or multiple lateral pelvic structures could be considered resectable with free surgical margins in 75-86% giving a promising survival outcome (Vizzielli et al., 2017; Vizzielli et al., 2019).

In this context, pre-operative, and intra- operative assessments before embarking on a pelvic exenteration, are of crucial importance to minimise harm to patients. In recent years, the advent of high-resolution MRI-scan and PET-CT scan can accurately define (if performed close to pelvic exenteration) the local infiltration and the potential presence of distant disease (Causa Andrieu et al., 2021). Obviously, the presence of suspicious distant metastasis (including inguino-femoral and para-aortic disease) might require a histologic assessment before performing the radical surgery. A multidisciplinary approach to MRI image review is of critical significance to correctly plan the site of resection, particularly in cases of laterally extended (endo)pelvic resections. Intra-operatively, the local assessment with examination under anaesthesia is also propaedeutic to the resection of the tumour and a diagnostic laparoscopy is advisable to exclude peritoneal carcinomatosis or tumour protruding through the peritoneum in the peritoneal cavity (e.g., From the pouch of Douglas). In our non-published experience of 78 patients who were identified as potential candidates for pelvic exenteration for gynaecological cancers between 2020 and 2022, 7 (9.0%) of them were aborted at the time of diagnostic laparoscopy for the previously described criteria. Lastly, the team should be prepared to deliver intra-operative radiation therapy or intra- operative positioning of brachytherapy catheters (to administer post-operative radiotherapy) where positive surgical margins are suspected or confirmed (Backes et al., 2014; Delara et al., 2021). It is in fact well established that negative surgical margins represent the most important prognostic factor in these patients (Sardain et al., 2015; Bizzarri et al., 2019).

When dealing with patients’ selection to minimally invasive pelvic exenteration, the triage process needs to be even more accurate. Despite the previously reported feasibility of a minimally invasive/ laparoscopic approach to laterally extended endopelvic resection (LEER) (Sozzi et al., 2019) and in large recurrent/persistent disease (Bizzarri et al., 2019), we believe that this procedure should be offered to patients with central pelvic recurrence/persistence and the tumour diameter of < 5 cm (Marnitz et al., 2006; Peiretti et al., 2012; Sardain et al., 2015). Obesity and medical morbidity preventing major laparotomy are potential patient’s characteristics favouring the minimally invasive approach. Table I shows the tumour and patient’s characteristics for selection to minimally invasive pelvic exenteration.

**Table I:** Tumour and patient’s characteristics to be considered for selection to minimally invasive pelvic exenteration (as compared to laparotomy).

Potential indications for minimally invasive pelvic exenteration	Potential indications for open pelvic exenteration
Major criteria:	Major criteria:
Small tumours (< 5 cm at pre-operative MRI scan)	Large tumours (≥ 5 cm at pre-operative MRI scan)
No pelvic side-wall involvement	Pelvic sidewall or lateral structures involvement
Morbidity of patient who could not tolerate major laparotomy	Patients able to tolerate major laparotomy
Consider in following categories:	Consider in following categories:
Palliative setting	Curative setting
BMI ≥ 30	BMI < 30

Palliative pelvic exenteration is performed in patients with no indications for curative exenteration. Curative pelvic exenteration is defined when clear margins can be pathologically ensured, and no distant metastases are found either intraabdominally or on the preoperative MRI/CT/ PET scan. An exenteration is defined as palliative in the presence of distant metastasis (including para-aortic and inguinofemoral lymph nodes), positive peritoneal washing or tumour perforation into the pouch of Douglas, as well as in cases when complete tumour removal was not possible. The indication for palliative pelvic exenteration is usually related to fistula symptoms or major bleeding not amenable to palliative radiotherapy (Schmidt et al., 2012; Guimarães et al., 2011).

We believe that a minimally invasive approach could be the procedure of choice for patients who are candidates for a palliative pelvic exenteration to provide symptom relief with the aim of achieving the least possible morbidity.

Concerning the survival outcomes associated with pelvic exenteration in gynaecological cancers, this is fairly heterogeneous. Table II demonstrates the most relevant studies in the literature reporting survival outcomes of pelvic exenterations for gynaecological cancers. Only studies reporting on open pelvic exenterations with more than 100 patients since 2012 have been reported. 5-year disease-free survival ranges from 33-61% and 5-year overall survival ranges from 27-41%. We must acknowledge that the heterogeneity of inclusion criteria of the different series is reflected in survival differences: in fact, the rate of palliative and laterally extended procedures is very different in the reported studies; similarly, the incidence of well-known prognostic factors after pelvic exenteration, such as involvement of surgical margins and lymph node metastasis is also quite dissimilar. Regarding the surgical approach, the oncological safety of minimally invasive surgery has been poorly studied and only a few studies have reported on this (Martínez et al., 2011; Puntambekar et al., 2016).

**Table II:** Outcomes of series reporting on open versus minimally invasive pelvic exenteration for gynaecologic malignancies (selected only open series with more than 100 patients since 2012).

Variables	Bizzarri et al. 2023	Puntambekar et al. 2016	Martinez et al. 2011	Graves et al. 2017	Westin et al. 2014	Chiantera et al. 2014	Schmidt et al. 2012	Baiocchi et al. 2012
Inclusion period	2010-2021	2005-2015	2000-2008	1998-2011	1993-2010	1998-2011	NR	1982-2010
Number of patients	117	74	43	313	160	167	282	107
Age (median)	60	50	58	52	55	51	50	56
Consecutive patients	Yes	No	Yes	No	Yes	Yes	No	No
Site of primary disease								
Cervix	66.7%	100%	67.4%	100%	53.8%	100%	100%	68.2%
Uterine corpus	22.2%	0	14.0%	0	9.4%	0	0	15.9%
Vagina	7.6%	0	0	0	23.8%	0	0	9.3%
Vulva	2.6%	0	11.6%	0	12.5%	0	0	6.5%
Others	0.8%	0	6.9%	0	0.6%	0	0	0
Time from primary treatment to pelvic exenteration	15 months	NR	23.7 months	NR	1.6 years	23.3 months	18 months	18.8 months
Surgical approach								
Laparotomy	66.7%	0	67.5%	100%	100%	100%	100%	100%
MIS	33.3%	100%	32.5%	0	0	0	0	0
Type of exenteration								
Anterior	59.8%	100%	53.5		21.3%	28.1%	4.9%	29.9%
Total	40.2%	0	27.9%	NR	68.8%	61.1%	92.9%	52.3%
Posterior	0	0	18.6%		10.0%	10.8%	2.1%	9.3%
LEER	20.5%	0	0	NR	0	NR	NR	9.3%
Surgical margins histology								
Negative	84.6%	100%	86%	NR	NR	72.5%	65%	92.1%
Micro	13.7%	0						
Macro	1.7%	0						
Pelvic Lymph Nodes positive histology	12.8%	56.7%	NR	53%		29.3%	21%	57%
Tumour diameter at histology (mm) (median)	30	55	NR	NR	NR	NR	NR	55
Adjuvant treatment	49.6%	56.7%	NR	53%		29.3%	21%	57%
Intent of PE								
Curative	88%	100%	100%	NR	100%	100%	47%	100%
Palliative	12%	0	0		0	0	53%**	0
Median follow-up	37 months	NR	45 months	NR	2.3 years	68 months	17 months	25.7 months
Disease-free survival	3y 27% Median 17 months	Recurrence rate 13.5%	2y 34-41% Median 14 months	NR	5y 33%	Mean 13 months	5y 61%	5y 35.8%
Overall survival	(CSS) 3y 37% Median 26 months	3y 45%; 5y 27%	2y 49-51% Median 18 months	Median 29.6 months	5y 40%	5y 38% Mean 19 months	5y 41%	5y 27.4%

*Inclusion criteria: curative with R0; **The exenteration was considered curative when clear margins were pathologically assured, and no distant metastases were found either intraabdominally or in the preoperative MRI/CT/PET scan. An exenteration was declared palliative in the presence of distant metastasis, positive peritoneal lavage, or perforation into the pouch of Douglas, as well as in cases when complete tumour removal was not possible.

Recently, we reported the survival analysis of propensity-matched series of open and minimally invasive pelvic exenteration performed for gynaecological cancers where no difference was found in disease-free or cancer-specific survival (Bizzarri et al., 2023). Patients undergoing minimally invasive surgery had a reduced rate of intra-operative blood transfusion and had a trend toward a better post-operative complication rate. We must acknowledge that palliative cases have been included in these series, with a consequent reduced survival in the entire cohort, comparing with other series of selected cases.

It is important to mention that a minimally invasive approach to pelvic exenteration should be performed by teams with a high level of skills in laparoscopic and robotic surgery in high-volume referral centres, possibly in the setting of clinical trials and following the basic principles of oncological surgery, thus avoiding cancer cell spillage, with careful specimen manipulation and resection of tumour-free tissues.

In conclusion, we believe that a minimally invasive approach to pelvic exenteration should be considered whenever possible, and gynaecological oncologists might want to consider it in selected cases like palliation and in the cases of small and central tumours. Such an approach should be avoided in large tumours (>5 cm) and in tumours with lateral sidewall involvement as achieving free surgical margins remains of crucial importance. Ideally, this approach should be used in the context of prospective clinical trials.
